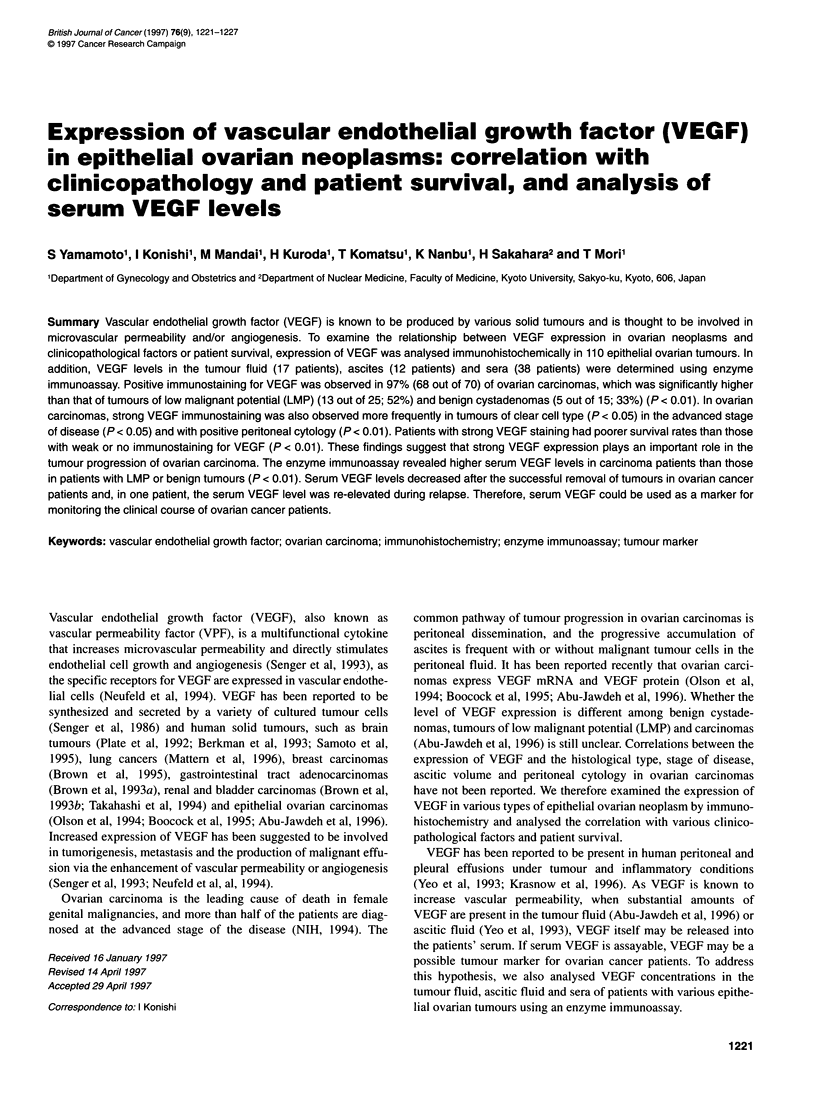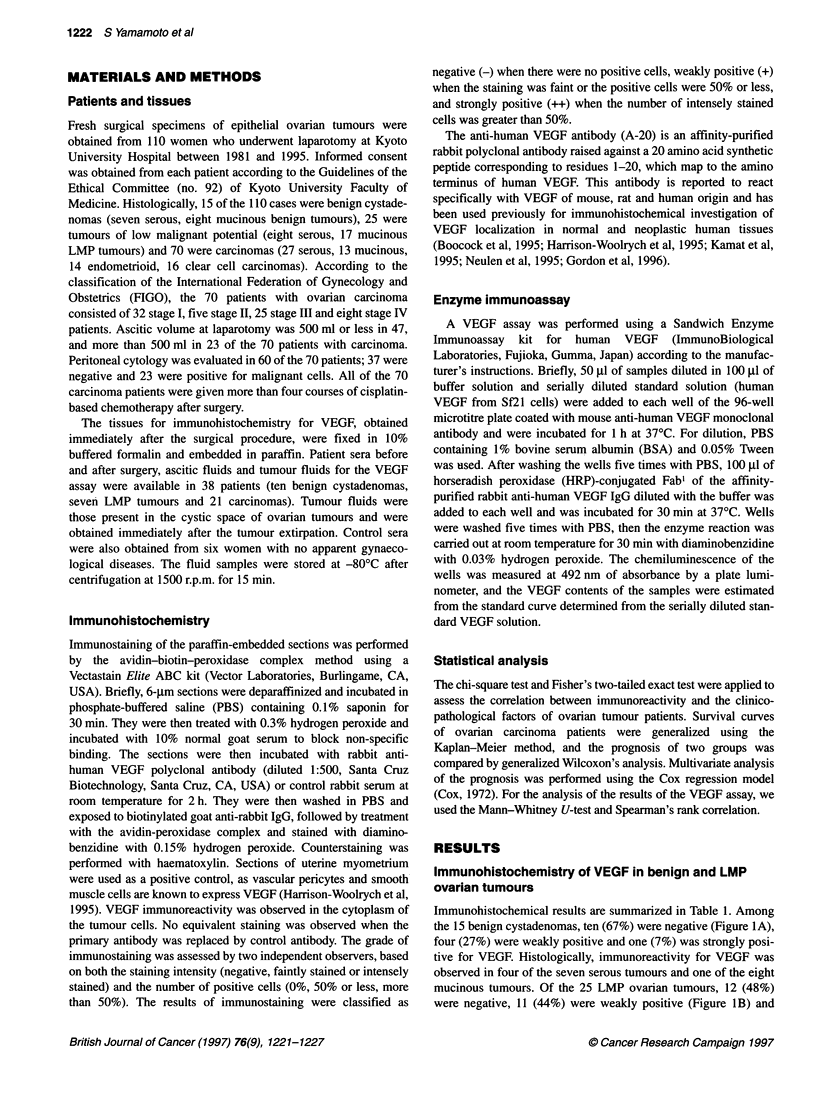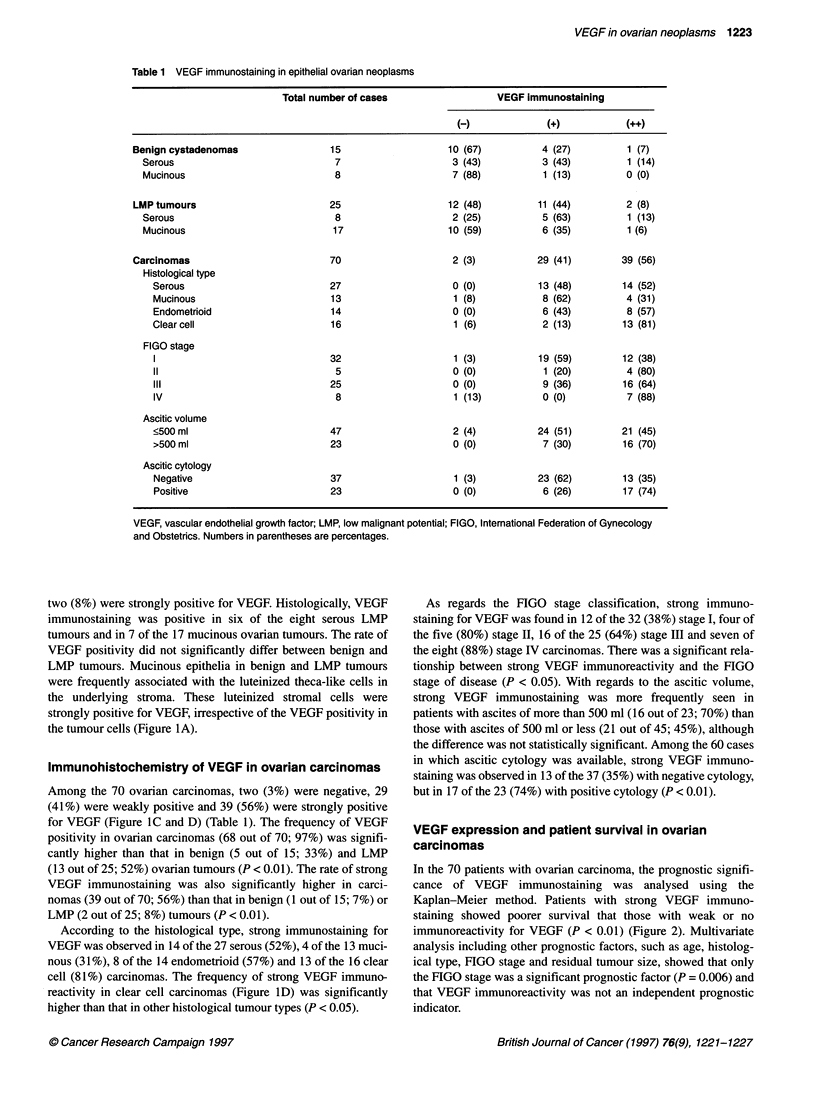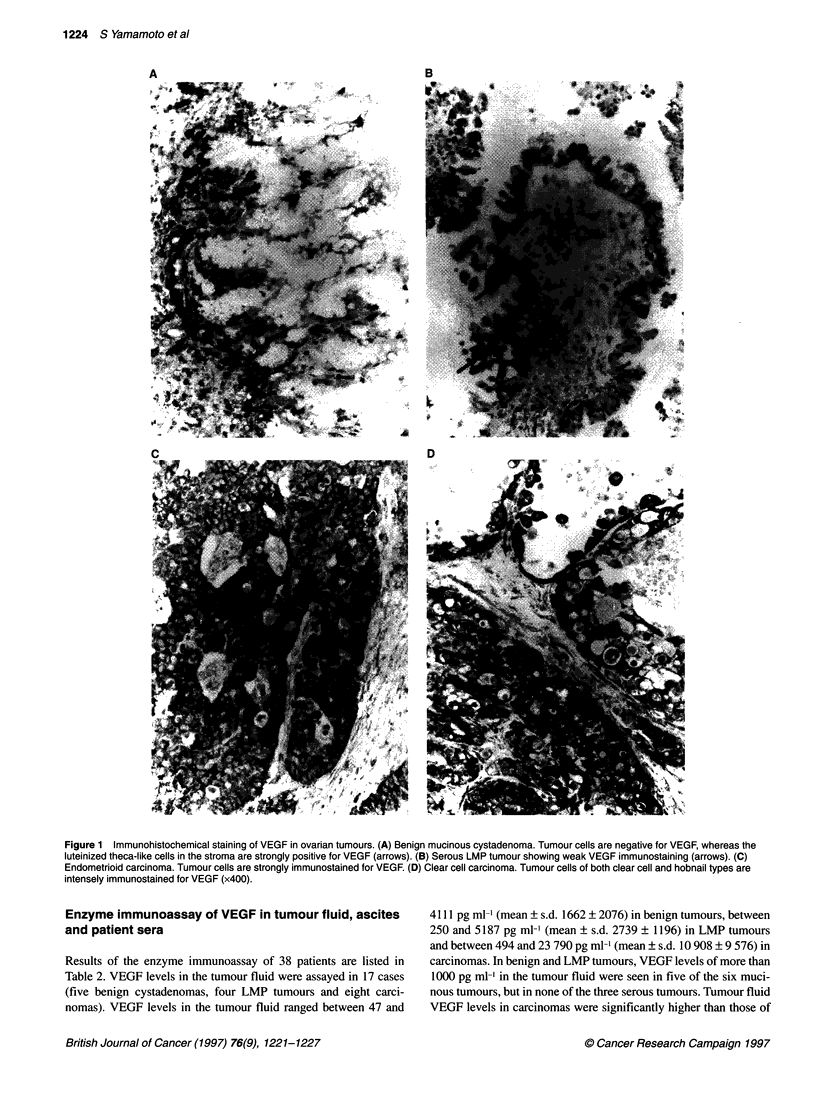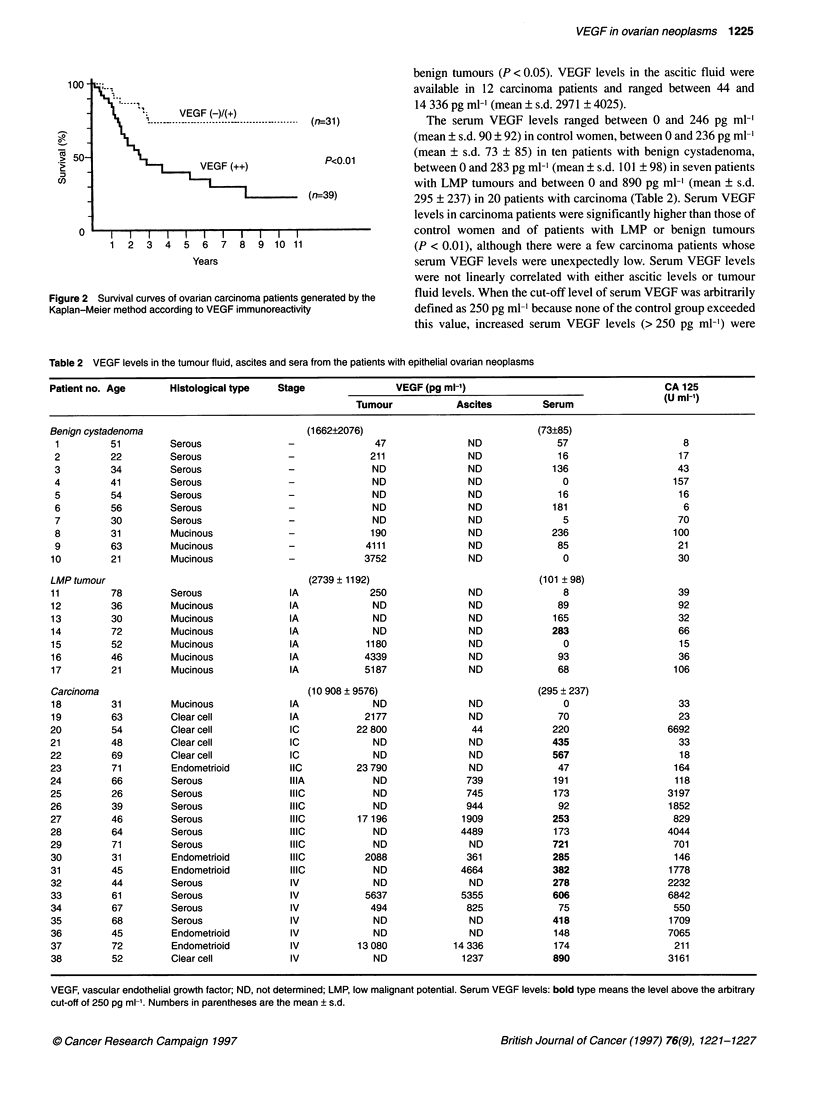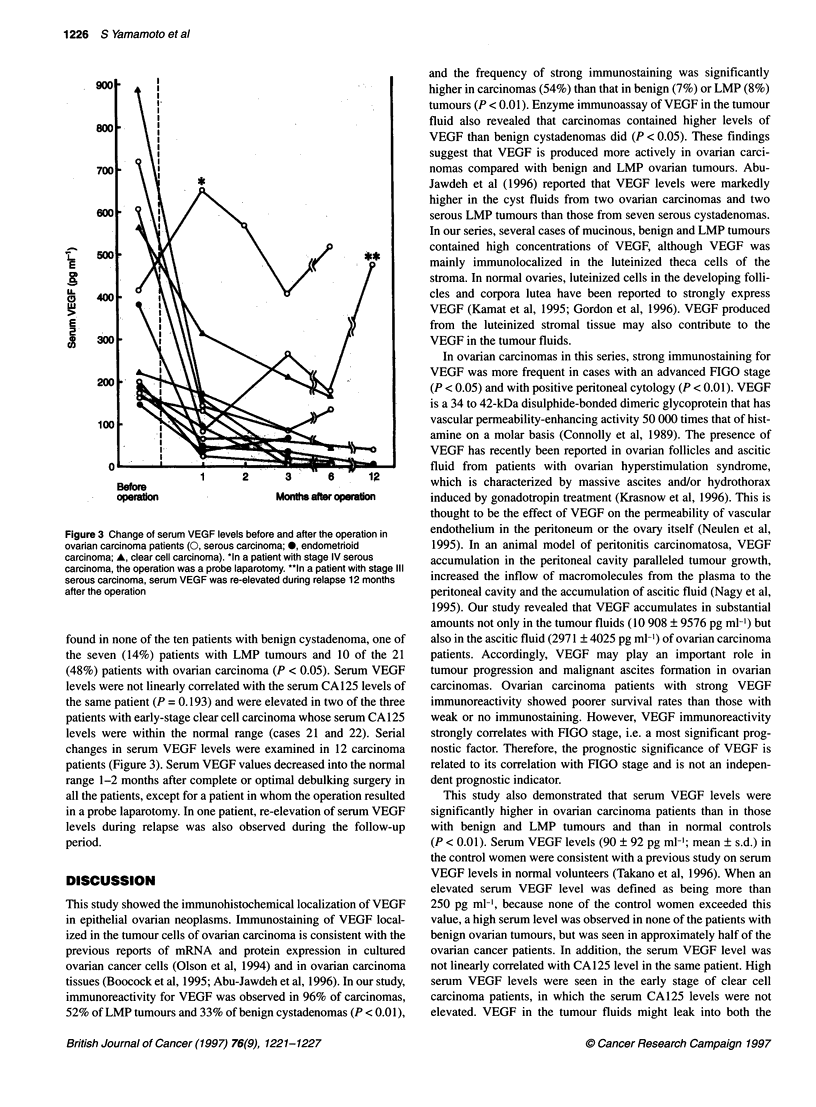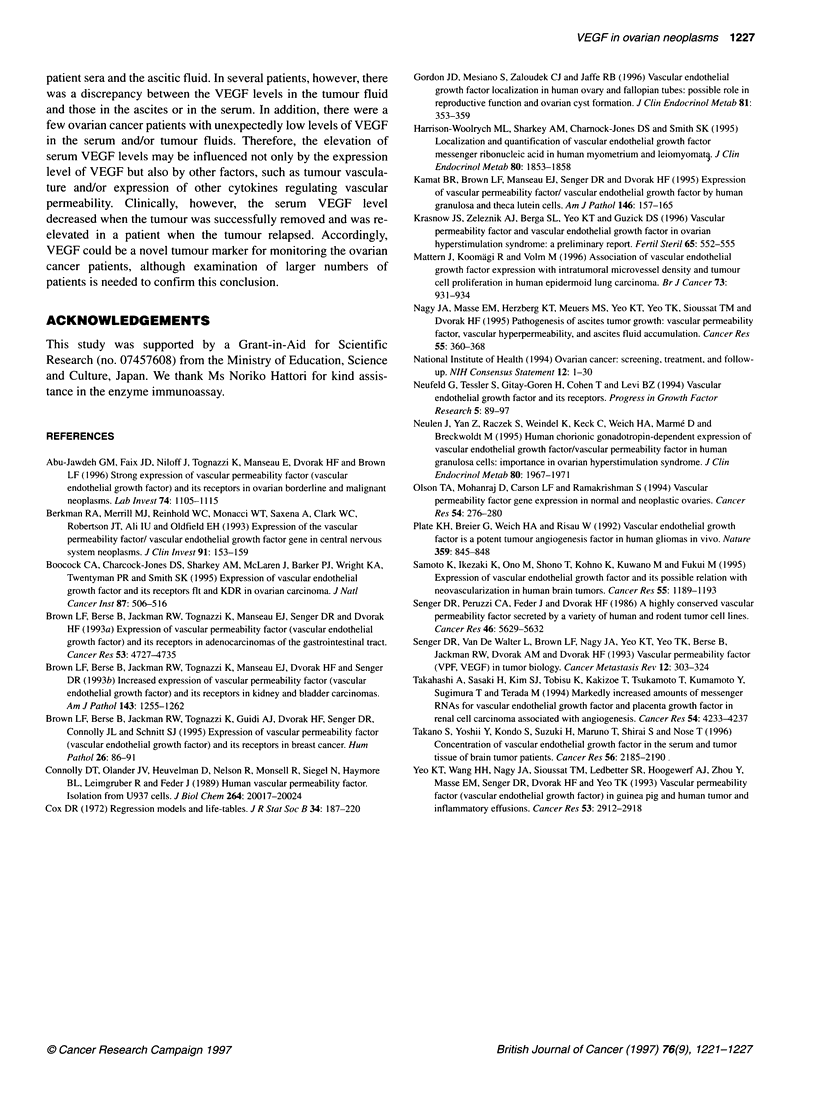# Expression of vascular endothelial growth factor (VEGF) in epithelial ovarian neoplasms: correlation with clinicopathology and patient survival, and analysis of serum VEGF levels.

**DOI:** 10.1038/bjc.1997.537

**Published:** 1997

**Authors:** S. Yamamoto, I. Konishi, M. Mandai, H. Kuroda, T. Komatsu, K. Nanbu, H. Sakahara, T. Mori

**Affiliations:** Department of Gynecology and Obstetrics, Faculty of Medicine, Kyoto University, Japan.

## Abstract

**Images:**


British Journal of Cancer (1997) 76(9), 1221–1227
© 1997 Cancer Research Campaign

### Expression of vascular endothelial growth factor (VEGF) in epithelial ovarian neoplasms: correlation with clinicopathology and patient survival, and analysis of serum VEGF levels

**S Yamamoto[1], I Konishi[1], M Mandai[1], H Kuroda[1], T Komatsu[1], K Nanbu[1], H Sakahara[2] and T Mori[1]**

[1]Department of Gynecology and Obstetrics and [2]Department of Nuclear Medicine, Faculty of Medicine, Kyoto University, Sakyo-ku, Kyoto, 606, Japan

**Summary** Vascular endothelial growth factor (VEGF) is known to be produced by various solid tumours and is thought to be involved in microvascular permeability and/or angiogenesis. To examine the relationship between VEGF expression in ovarian neoplasms and clinicopathological factors or patient survival, expression of VEGF was analysed immunohistochemically in 110 epithelial ovarian tumours. In addition, VEGF levels in the tumour fluid (17 patients), ascites (12 patients) and sera (38 patients) were determined using enzyme immunoassay. Positive immunostaining for VEGF was observed in 97% (68 out of 70) of ovarian carcinomas, which was significantly higher than that of tumours of low malignant potential (LMP) (13 out of 25; 52%) and benign cystadenomas (5 out of 15; 33%) ($P < 0.01$). In ovarian carcinomas, strong VEGF immunostaining was also observed more frequently in tumours of clear cell type ($P < 0.05$) in the advanced stage of disease ($P < 0.05$) and with positive peritoneal cytology ($P < 0.01$). Patients with strong VEGF staining had poorer survival rates than those with weak or no immunostaining for VEGF ($P < 0.01$). These findings suggest that strong VEGF expression plays an important role in the tumour progression of ovarian carcinoma. The enzyme immunoassay revealed higher serum VEGF levels in carcinoma patients than those in patients with LMP or benign tumours ($P < 0.01$). Serum VEGF levels decreased after the successful removal of tumours in ovarian cancer patients and, in one patient, the serum VEGF level was re-elevated during relapse. Therefore, serum VEGF could be used as a marker for monitoring the clinical course of ovarian cancer patients.

**Keywords:** vascular endothelial growth factor; ovarian carcinoma; immunohistochemistry; enzyme immunoassay; tumour marker

Vascular endothelial growth factor (VEGF), also known as vascular permeability factor (VPF), is a multifunctional cytokine that increases microvascular permeability and directly stimulates endothelial cell growth and angiogenesis (Senger et al, 1993), as the specific receptors for VEGF are expressed in vascular endothelial cells (Neufeld et al, 1994). VEGF has been reported to be synthesized and secreted by a variety of cultured tumour cells (Senger et al, 1986) and human solid tumours, such as brain tumours (Plate et al, 1992; Berkman et al, 1993; Samoto et al, 1995), lung cancers (Mattern et al, 1996), breast carcinomas (Brown et al, 1995), gastrointestinal tract adenocarcinomas (Brown et al, 1993*a*), renal and bladder carcinomas (Brown et al, 1993*b*; Takahashi et al, 1994) and epithelial ovarian carcinomas (Olson et al, 1994; Boocock et al, 1995; Abu-Jawdeh et al, 1996). Increased expression of VEGF has been suggested to be involved in tumorigenesis, metastasis and the production of malignant effusion via the enhancement of vascular permeability or angiogenesis (Senger et al, 1993; Neufeld et al, al, 1994).

Ovarian carcinoma is the leading cause of death in female genital malignancies, and more than half of the patients are diagnosed at the advanced stage of the disease (NIH, 1994). The common pathway of tumour progression in ovarian carcinomas is peritoneal dissemination, and the progressive accumulation of ascites is frequent with or without malignant tumour cells in the peritoneal fluid. It has been reported recently that ovarian carcinomas express VEGF mRNA and VEGF protein (Olson et al, 1994; Boocock et al, 1995; Abu-Jawdeh et al, 1996). Whether the level of VEGF expression is different among benign cystadenomas, tumours of low malignant potential (LMP) and carcinomas (Abu-Jawdeh et al, 1996) is still unclear. Correlations between the expression of VEGF and the histological type, stage of disease, ascitic volume and peritoneal cytology in ovarian carcinomas have not been reported. We therefore examined the expression of VEGF in various types of epithelial ovarian neoplasm by immunohistochemistry and analysed the correlation with various clinicopathological factors and patient survival.

VEGF has been reported to be present in human peritoneal and pleural effusions under tumour and inflammatory conditions (Yeo et al, 1993; Krasnow et al, 1996). As VEGF is known to increase vascular permeability, when substantial amounts of VEGF are present in the tumour fluid (Abu-Jawdeh et al, 1996) or ascitic fluid (Yeo et al, 1993), VEGF itself may be released into the patients' serum. If serum VEGF is assayable, VEGF may be a possible tumour marker for ovarian cancer patients. To address this hypothesis, we also analysed VEGF concentrations in the tumour fluid, ascitic fluid and sera of patients with various epithelial ovarian tumours using an enzyme immunoassay.

*Received 16 January 1997*
*Revised 14 April 1997*
*Accepted 29 April 1997*

*Correspondence to:* I Konishi

### MATERIALS AND METHODS

### Patients and tissues

Fresh surgical specimens of epithelial ovarian tumours were obtained from 110 women who underwent laparotomy at Kyoto University Hospital between 1981 and 1995. Informed consent was obtained from each patient according to the Guidelines of the Ethical Committee (no. 92) of Kyoto University Faculty of Medicine. Histologically, 15 of the 110 cases were benign cystadenomas (seven serous, eight mucinous benign tumours), 25 were tumours of low malignant potential (eight serous, 17 mucinous LMP tumours) and 70 were carcinomas (27 serous, 13 mucinous, 14 endometrioid, 16 clear cell carcinomas). According to the classification of the International Federation of Gynecology and Obstetrics (FIGO), the 70 patients with ovarian carcinoma consisted of 32 stage I, five stage II, 25 stage III and eight stage IV patients. Ascitic volume at laparotomy was 500 ml or less in 47, and more than 500 ml in 23 of the 70 patients with carcinoma. Peritoneal cytology was evaluated in 60 of the 70 patients; 37 were negative and 23 were positive for malignant cells. All of the 70 carcinoma patients were given more than four courses of cisplatin-based chemotherapy after surgery.

The tissues for immunohistochemistry for VEGF, obtained immediately after the surgical procedure, were fixed in 10% buffered formalin and embedded in paraffin. Patient sera before and after surgery, ascitic fluids and tumour fluids for the VEGF assay were available in 38 patients (ten benign cystadenomas, seven LMP tumours and 21 carcinomas). Tumour fluids were those present in the cystic space of ovarian tumours and were obtained immediately after the tumour extirpation. Control sera were also obtained from six women with no apparent gynaecological diseases. The fluid samples were stored at –80°C after centrifugation at 1500 r.p.m. for 15 min.

### Immunohistochemistry

Immunostaining of the paraffin-embedded sections was performed by the avidin–biotin–peroxidase complex method using a Vectastain *Elite* ABC kit (Vector Laboratories, Burlingame, CA, USA). Briefly, 6-μm sections were deparaffinized and incubated in phosphate-buffered saline (PBS) containing 0.1% saponin for 30 min. They were then treated with 0.3% hydrogen peroxide and incubated with 10% normal goat serum to block non-specific binding. The sections were then incubated with rabbit anti-human VEGF polyclonal antibody (diluted 1:500, Santa Cruz Biotechnology, Santa Cruz, CA, USA) or control rabbit serum at room temperature for 2 h. They were then washed in PBS and exposed to biotinylated goat anti-rabbit IgG, followed by treatment with the avidin-peroxidase complex and stained with diaminobenzidine with 0.15% hydrogen peroxide. Counterstaining was performed with haematoxylin. Sections of uterine myometrium were used as a positive control, as vascular pericytes and smooth muscle cells are known to express VEGF (Harrison-Woolrych et al, 1995). VEGF immunoreactivity was observed in the cytoplasm of the tumour cells. No equivalent staining was observed when the primary antibody was replaced by control antibody. The grade of immunostaining was assessed by two independent observers, based on both the staining intensity (negative, faintly stained or intensely stained) and the number of positive cells (0%, 50% or less, more than 50%). The results of immunostaining were classified as negative (–) when there were no positive cells, weakly positive (+) when the staining was faint or the positive cells were 50% or less, and strongly positive (++) when the number of intensely stained cells was greater than 50%.

The anti-human VEGF antibody (A-20) is an affinity-purified rabbit polyclonal antibody raised against a 20 amino acid synthetic peptide corresponding to residues 1–20, which map to the amino terminus of human VEGF. This antibody is reported to react specifically with VEGF of mouse, rat and human origin and has been used previously for immunohistochemical investigation of VEGF localization in normal and neoplastic human tissues (Boocock et al, 1995; Harrison-Woolrych et al, 1995; Kamat et al, 1995; Neulen et al, 1995; Gordon et al, 1996).

### Enzyme immunoassay

A VEGF assay was performed using a Sandwich Enzyme Immunoassay kit for human VEGF (ImmunoBiological Laboratories, Fujioka, Gumma, Japan) according to the manufacturer's instructions. Briefly, 50 μl of samples diluted in 100 μl of buffer solution and serially diluted standard solution (human VEGF from Sf21 cells) were added to each well of the 96-well microtitre plate coated with mouse anti-human VEGF monoclonal antibody and were incubated for 1 h at 37°C. For dilution, PBS containing 1% bovine serum albumin (BSA) and 0.05% Tween was used. After washing the wells five times with PBS, 100 μl of horseradish peroxidase (HRP)-conjugated Fab[1] of the affinity-purified rabbit anti-human VEGF IgG diluted with the buffer was added to each well and was incubated for 30 min at 37°C. Wells were washed five times with PBS, then the enzyme reaction was carried out at room temperature for 30 min with diaminobenzidine with 0.03% hydrogen peroxide. The chemiluminescence of the wells was measured at 492 nm of absorbance by a plate luminometer, and the VEGF contents of the samples were estimated from the standard curve determined from the serially diluted standard VEGF solution.

### Statistical analysis

The chi-square test and Fisher's two-tailed exact test were applied to assess the correlation between immunoreactivity and the clinicopathological factors of ovarian tumour patients. Survival curves of ovarian carcinoma patients were generalized using the Kaplan–Meier method, and the prognosis of two groups was compared by generalized Wilcoxon's analysis. Multivariate analysis of the prognosis was performed using the Cox regression model (Cox, 1972). For the analysis of the results of the VEGF assay, we used the Mann–Whitney *U*-test and Spearman's rank correlation.

### RESULTS

### Immunohistochemistry of VEGF in benign and LMP ovarian tumours

Immunohistochemical results are summarized in Table 1. Among the 15 benign cystadenomas, ten (67%) were negative (Figure 1A), four (27%) were weakly positive and one (7%) was strongly positive for VEGF. Histologically, immunoreactivity for VEGF was observed in four of the seven serous tumours and one of the eight mucinous tumours. Of the 25 LMP ovarian tumours, 12 (48%) were negative, 11 (44%) were weakly positive (Figure 1B) and

© Cancer Research Campaign 1997

**Table 1** VEGF immunostaining in epithelial ovarian neoplasms

| | Total number of cases | VEGF immunostaining | | |
|---|---|---|---|---|
| | | (–) | (+) | (++) |
| **Benign cystadenomas** | 15 | 10 (67) | 4 (27) | 1 (7) |
| Serous | 7 | 3 (43) | 3 (43) | 1 (14) |
| Mucinous | 8 | 7 (88) | 1 (13) | 0 (0) |
| **LMP tumours** | 25 | 12 (48) | 11 (44) | 2 (8) |
| Serous | 8 | 2 (25) | 5 (63) | 1 (13) |
| Mucinous | 17 | 10 (59) | 6 (35) | 1 (6) |
| **Carcinomas** | 70 | 2 (3) | 29 (41) | 39 (56) |
| Histological type | | | | |
| Serous | 27 | 0 (0) | 13 (48) | 14 (52) |
| Mucinous | 13 | 1 (8) | 8 (62) | 4 (31) |
| Endometrioid | 14 | 0 (0) | 6 (43) | 8 (57) |
| Clear cell | 16 | 1 (6) | 2 (13) | 13 (81) |
| FIGO stage | | | | |
| I | 32 | 1 (3) | 19 (59) | 12 (38) |
| II | 5 | 0 (0) | 1 (20) | 4 (80) |
| III | 25 | 0 (0) | 9 (36) | 16 (64) |
| IV | 8 | 1 (13) | 0 (0) | 7 (88) |
| Ascitic volume | | | | |
| ≤500 ml | 47 | 2 (4) | 24 (51) | 21 (45) |
| >500 ml | 23 | 0 (0) | 7 (30) | 16 (70) |
| Ascitic cytology | | | | |
| Negative | 37 | 1 (3) | 23 (62) | 13 (35) |
| Positive | 23 | 0 (0) | 6 (26) | 17 (74) |

VEGF, vascular endothelial growth factor; LMP, low malignant potential; FIGO, International Federation of Gynecology and Obstetrics. Numbers in parentheses are percentages.

two (8%) were strongly positive for VEGF. Histologically, VEGF immunostaining was positive in six of the eight serous LMP tumours and in 7 of the 17 mucinous ovarian tumours. The rate of VEGF positivity did not significantly differ between benign and LMP tumours. Mucinous epithelia in benign and LMP tumours were frequently associated with the luteinized theca-like cells in the underlying stroma. These luteinized stromal cells were strongly positive for VEGF, irrespective of the VEGF positivity in the tumour cells (Figure 1A).

### Immunohistochemistry of VEGF in ovarian carcinomas

Among the 70 ovarian carcinomas, two (3%) were negative, 29 (41%) were weakly positive and 39 (56%) were strongly positive for VEGF (Figure 1C and D) (Table 1). The frequency of VEGF positivity in ovarian carcinomas (68 out of 70; 97%) was significantly higher than that in benign (5 out of 15; 33%) and LMP (13 out of 25; 52%) ovarian tumours ($P < 0.01$). The rate of strong VEGF immunostaining was also significantly higher in carcinomas (39 out of 70; 56%) than that in benign (1 out of 15; 7%) or LMP (2 out of 25; 8%) tumours ($P < 0.01$).

According to the histological type, strong immunostaining for VEGF was observed in 14 of the 27 serous (52%), 4 of the 13 mucinous (31%), 8 of the 14 endometrioid (57%) and 13 of the 16 clear cell (81%) carcinomas. The frequency of strong VEGF immunoreactivity in clear cell carcinomas (Figure 1D) was significantly higher than that in other histological tumour types ($P < 0.05$).

As regards the FIGO stage classification, strong immunostaining for VEGF was found in 12 of the 32 (38%) stage I, four of the five (80%) stage II, 16 of the 25 (64%) stage III and seven of the eight (88%) stage IV carcinomas. There was a significant relationship between strong VEGF immunoreactivity and the FIGO stage of disease ($P < 0.05$). With regards to the ascitic volume, strong VEGF immunostaining was more frequently seen in patients with ascites of more than 500 ml (16 out of 23; 70%) than those with ascites of 500 ml or less (21 out of 45; 45%), although the difference was not statistically significant. Among the 60 cases in which ascitic cytology was available, strong VEGF immunostaining was observed in 13 of the 37 (35%) with negative cytology, but in 17 of the 23 (74%) with positive cytology ($P < 0.01$).

### VEGF expression and patient survival in ovarian carcinomas

In the 70 patients with ovarian carcinoma, the prognostic significance of VEGF immunostaining was analysed using the Kaplan–Meier method. Patients with strong VEGF immunostaining showed poorer survival that those with weak or no immunoreactivity for VEGF ($P < 0.01$) (Figure 2). Multivariate analysis including other prognostic factors, such as age, histological type, FIGO stage and residual tumour size, showed that only the FIGO stage was a significant prognostic factor ($P = 0.006$) and that VEGF immunoreactivity was not an independent prognostic indicator.

© Cancer Research Campaign 1997 

**Figure 1** Immunohistochemical staining of VEGF in ovarian tumours. (**A**) Benign mucinous cystadenoma. Tumour cells are negative for VEGF, whereas the luteinized theca-like cells in the stroma are strongly positive for VEGF (arrows). (**B**) Serous LMP tumour showing weak VEGF immunostaining (arrows). (**C**) Endometrioid carcinoma. Tumour cells are strongly immunostained for VEGF. (**D**) Clear cell carcinoma. Tumour cells of both clear cell and hobnail types are intensely immunostained for VEGF (×400).

### Enzyme immunoassay of VEGF in tumour fluid, ascites and patient sera

Results of the enzyme immunoassay of 38 patients are listed in Table 2. VEGF levels in the tumour fluid were assayed in 17 cases (five benign cystadenomas, four LMP tumours and eight carcinomas). VEGF levels in the tumour fluid ranged between 47 and 4111 pg $ml^{-1}$ (mean ± s.d. 1662 ± 2076) in benign tumours, between 250 and 5187 pg $ml^{-1}$ (mean ± s.d. 2739 ± 1196) in LMP tumours and between 494 and 23 790 pg $ml^{-1}$ (mean ± s.d. 10 908 ± 9 576) in carcinomas. In benign and LMP tumours, VEGF levels of more than 1000 pg $ml^{-1}$ in the tumour fluid were seen in five of the six mucinous tumours, but in none of the three serous tumours. Tumour fluid VEGF levels in carcinomas were significantly higher than those of

© Cancer Research Campaign 1997

**Figure 2** Survival curves of ovarian carcinoma patients generated by the Kaplan–Meier method according to VEGF immunoreactivity benign tumours ($P < 0.05$). VEGF levels in the ascitic fluid were available in 12 carcinoma patients and ranged between 44 and 14 336 pg ml$^{-1}$ (mean ± s.d. 2971 ± 4025).

The serum VEGF levels ranged between 0 and 246 pg ml$^{-1}$ (mean ± s.d. 90 ± 92) in control women, between 0 and 236 pg ml$^{-1}$ (mean ± s.d. 73 ± 85) in ten patients with benign cystadenoma, between 0 and 283 pg ml$^{-1}$ (mean ± s.d. 101 ± 98) in seven patients with LMP tumours and between 0 and 890 pg ml$^{-1}$ (mean ± s.d. 295 ± 237) in 20 patients with carcinoma (Table 2). Serum VEGF levels in carcinoma patients were significantly higher than those of control women and of patients with LMP or benign tumours ($P < 0.01$), although there were a few carcinoma patients whose serum VEGF levels were unexpectedly low. Serum VEGF levels were not linearly correlated with either ascitic levels or tumour fluid levels. When the cut-off level of serum VEGF was arbitrarily defined as 250 pg ml$^{-1}$ because none of the control group exceeded this value, increased serum VEGF levels (> 250 pg ml$^{-1}$) were

**Table 2** VEGF levels in the tumour fluid, ascites and sera from the patients with epithelial ovarian neoplasms

| Patient no. | Age | Histological type | Stage | VEGF (pg ml$^{-1}$) | | | CA 125 (U ml$^{-1}$) |
|---|---|---|---|---|---|---|---|
| | | | | Tumour | Ascites | Serum | |
| *Benign cystadenoma* | | | | (1662±2076) | | (73±85) | |
| 1 | 51 | Serous | – | 47 | ND | 57 | 8 |
| 2 | 22 | Serous | – | 211 | ND | 16 | 17 |
| 3 | 34 | Serous | – | ND | ND | 136 | 43 |
| 4 | 41 | Serous | – | ND | ND | 0 | 157 |
| 5 | 54 | Serous | – | ND | ND | 16 | 16 |
| 6 | 56 | Serous | – | ND | ND | 181 | 6 |
| 7 | 30 | Serous | – | ND | ND | 5 | 70 |
| 8 | 31 | Mucinous | – | 190 | ND | 236 | 100 |
| 9 | 63 | Mucinous | – | 4111 | ND | 85 | 21 |
| 10 | 21 | Mucinous | – | 3752 | ND | 0 | 30 |
| *LMP tumour* | | | | (2739 ± 1192) | | (101 ± 98) | |
| 11 | 78 | Serous | IA | 250 | ND | 8 | 39 |
| 12 | 36 | Mucinous | IA | ND | ND | 89 | 92 |
| 13 | 30 | Mucinous | IA | ND | ND | 165 | 32 |
| 14 | 72 | Mucinous | IA | ND | ND | **283** | 66 |
| 15 | 52 | Mucinous | IA | 1180 | ND | 0 | 15 |
| 16 | 46 | Mucinous | IA | 4339 | ND | 93 | 36 |
| 17 | 21 | Mucinous | IA | 5187 | ND | 68 | 106 |
| *Carcinoma* | | | | (10 908 ± 9576) | | (295 ± 237) | |
| 18 | 31 | Mucinous | IA | ND | ND | 0 | 33 |
| 19 | 63 | Clear cell | IA | 2177 | ND | 70 | 23 |
| 20 | 54 | Clear cell | IC | 22 800 | 44 | 220 | 6692 |
| 21 | 48 | Clear cell | IC | ND | ND | **435** | 33 |
| 22 | 69 | Clear cell | IC | ND | ND | **567** | 18 |
| 23 | 71 | Endometrioid | IIC | 23 790 | ND | 47 | 164 |
| 24 | 66 | Serous | IIIA | ND | 739 | 191 | 118 |
| 25 | 26 | Serous | IIIC | ND | 745 | 173 | 3197 |
| 26 | 39 | Serous | IIIC | ND | 944 | 92 | 1852 |
| 27 | 46 | Serous | IIIC | 17 196 | 1909 | **253** | 829 |
| 28 | 64 | Serous | IIIC | ND | 4489 | 173 | 4044 |
| 29 | 71 | Serous | IIIC | ND | ND | **721** | 701 |
| 30 | 31 | Endometrioid | IIIC | 2088 | 361 | **285** | 146 |
| 31 | 45 | Endometrioid | IIIC | ND | 4664 | **382** | 1778 |
| 32 | 44 | Serous | IV | ND | ND | **278** | 2232 |
| 33 | 61 | Serous | IV | 5637 | 5355 | **606** | 6842 |
| 34 | 67 | Serous | IV | 494 | 825 | 75 | 550 |
| 35 | 68 | Serous | IV | ND | ND | **418** | 1709 |
| 36 | 45 | Endometrioid | IV | ND | ND | 148 | 7065 |
| 37 | 72 | Endometrioid | IV | 13 080 | 14 336 | 174 | 211 |
| 38 | 52 | Clear cell | IV | ND | 1237 | **890** | 3161 |

VEGF, vascular endothelial growth factor; ND, not determined; LMP, low malignant potential. Serum VEGF levels: **bold** type means the level above the arbitrary cut-off of 250 pg ml$^{-1}$. Numbers in parentheses are the mean ± s.d.

© Cancer Research Campaign 1997

**Figure 3** Change of serum VEGF levels before and after the operation in ovarian carcinoma patients (○, serous carcinoma; ●, endometrioid carcinoma; ▲, clear cell carcinoma). *In a patient with stage IV serous carcinoma, the operation was a probe laparotomy. **In a patient with stage III serous carcinoma, serum VEGF was re-elevated during relapse 12 months after the operation found in none of the ten patients with benign cystadenoma, one of the seven (14%) patients with LMP tumours and 10 of the 21 (48%) patients with ovarian carcinoma ($P < 0.05$). Serum VEGF levels were not linearly correlated with the serum CA125 levels of the same patient ($P = 0.193$) and were elevated in two of the three patients with early-stage clear cell carcinoma whose serum CA125 levels were within the normal range (cases 21 and 22). Serial changes in serum VEGF levels were examined in 12 carcinoma patients (Figure 3). Serum VEGF values decreased into the normal range 1–2 months after complete or optimal debulking surgery in all the patients, except for a patient in whom the operation resulted in a probe laparotomy. In one patient, re-elevation of serum VEGF levels during relapse was also observed during the follow-up period.

### DISCUSSION

This study showed the immunohistochemical localization of VEGF in epithelial ovarian neoplasms. Immunostaining of VEGF localized in the tumour cells of ovarian carcinoma is consistent with the previous reports of mRNA and protein expression in cultured ovarian cancer cells (Olson et al, 1994) and in ovarian carcinoma tissues (Boocock et al, 1995; Abu-Jawdeh et al, 1996). In our study, immunoreactivity for VEGF was observed in 96% of carcinomas, 52% of LMP tumours and 33% of benign cystadenomas ($P < 0.01$), and the frequency of strong immunostaining was significantly higher in carcinomas (54%) than that in benign (7%) or LMP (8%) tumours ($P < 0.01$). Enzyme immunoassay of VEGF in the tumour fluid also revealed that carcinomas contained higher levels of VEGF than benign cystadenomas did ($P < 0.05$). These findings suggest that VEGF is produced more actively in ovarian carcinomas compared with benign and LMP ovarian tumours. Abu-Jawdeh et al (1996) reported that VEGF levels were markedly higher in the cyst fluids from two ovarian carcinomas and two serous LMP tumours than those from seven serous cystadenomas. In our series, several cases of mucinous, benign and LMP tumours contained high concentrations of VEGF, although VEGF was mainly immunolocalized in the luteinized theca cells of the stroma. In normal ovaries, luteinized cells in the developing follicles and corpora lutea have been reported to strongly express VEGF (Kamat et al, 1995; Gordon et al, 1996). VEGF produced from the luteinized stromal tissue may also contribute to the VEGF in the tumour fluids.

In ovarian carcinomas in this series, strong immunostaining for VEGF was more frequent in cases with an advanced FIGO stage ($P < 0.05$) and with positive peritoneal cytology ($P < 0.01$). VEGF is a 34 to 42-kDa disulphide-bonded dimeric glycoprotein that has vascular permeability-enhancing activity 50 000 times that of histamine on a molar basis (Connolly et al, 1989). The presence of VEGF has recently been reported in ovarian follicles and ascitic fluid from patients with ovarian hyperstimulation syndrome, which is characterized by massive ascites and/or hydrothorax induced by gonadotropin treatment (Krasnow et al, 1996). This is thought to be the effect of VEGF on the permeability of vascular endothelium in the peritoneum or the ovary itself (Neulen et al, 1995). In an animal model of peritonitis carcinomatosa, VEGF accumulation in the peritoneal cavity paralleled tumour growth, increased the inflow of macromolecules from the plasma to the peritoneal cavity and the accumulation of ascitic fluid (Nagy et al, 1995). Our study revealed that VEGF accumulates in substantial amounts not only in the tumour fluids (10 908 ± 9576 pg ml$^{-1}$) but also in the ascitic fluid (2971 ± 4025 pg ml$^{-1}$) of ovarian carcinoma patients. Accordingly, VEGF may play an important role in tumour progression and malignant ascites formation in ovarian carcinomas. Ovarian carcinoma patients with strong VEGF immunoreactivity showed poorer survival rates than those with weak or no immunostaining. However, VEGF immunoreactivity strongly correlates with FIGO stage, i.e. a most significant prognostic factor. Therefore, the prognostic significance of VEGF is related to its correlation with FIGO stage and is not an independent prognostic indicator.

This study also demonstrated that serum VEGF levels were significantly higher in ovarian carcinoma patients than in those with benign and LMP tumours and than in normal controls ($P < 0.01$). Serum VEGF levels (90 ± 92 pg ml$^{-1}$; mean ± s.d.) in the control women were consistent with a previous study on serum VEGF levels in normal volunteers (Takano et al, 1996). When an elevated serum VEGF level was defined as being more than 250 pg ml$^{-1}$, because none of the control women exceeded this value, a high serum level was observed in none of the patients with benign ovarian tumours, but was seen in approximately half of the ovarian cancer patients. In addition, the serum VEGF level was not linearly correlated with CA125 level in the same patient. High serum VEGF levels were seen in the early stage of clear cell carcinoma patients, in which the serum CA125 levels were not elevated. VEGF in the tumour fluids might leak into both the

© Cancer Research Campaign 1997

patient sera and the ascitic fluid. In several patients, however, there was a discrepancy between the VEGF levels in the tumour fluid and those in the ascites or in the serum. In addition, there were a few ovarian cancer patients with unexpectedly low levels of VEGF in the serum and/or tumour fluids. Therefore, the elevation of serum VEGF levels may be influenced not only by the expression level of VEGF but also by other factors, such as tumour vasculature and/or expression of other cytokines regulating vascular permeability. Clinically, however, the serum VEGF level decreased when the tumour was successfully removed and was re-elevated in a patient when the tumour relapsed. Accordingly, VEGF could be a novel tumour marker for monitoring the ovarian cancer patients, although examination of larger numbers of patients is needed to confirm this conclusion.

### ACKNOWLEDGEMENTS

This study was supported by a Grant-in-Aid for Scientific Research (no. 07457608) from the Ministry of Education, Science and Culture, Japan. We thank Ms Noriko Hattori for kind assistance in the enzyme immunoassay.

### REFERENCES

Abu-Jawdeh GM, Faix JD, Niloff J, Tognazzi K, Manseau E, Dvorak HF and Brown LF (1996) Strong expression of vascular permeability factor (vascular endothelial growth factor) and its receptors in ovarian borderline and malignant neoplasms. *Lab Invest* **74**: 1105–1115

Berkman RA, Merrill MJ, Reinhold WC, Monacci WT, Saxena A, Clark WC, Robertson JT, Ali IU and Oldfield EH (1993) Expression of the vascular permeability factor/ vascular endothelial growth factor gene in central nervous system neoplasms. *J Clin Invest* **91**: 153–159

Boocock CA, Charcock-Jones DS, Sharkey AM, McLaren J, Barker PJ, Wright KA, Twentyman PR and Smith SK (1995) Expression of vascular endothelial growth factor and its receptors flt and KDR in ovarian carcinoma. *J Natl Cancer Inst* **87**: 506–516

Brown LF, Berse B, Jackman RW, Tognazzi K, Manseau EJ, Senger DR and Dvorak HF (1993*a*) Expression of vascular permeability factor (vascular endothelial growth factor) and its receptors in adenocarcinomas of the gastrointestinal tract. *Cancer Res* **53**: 4727–4735

Brown LF, Berse B, Jackman RW, Tognazzi K, Manseau EJ, Dvorak HF and Senger DR (1993*b*) Increased expression of vascular permeability factor (vascular endothelial growth factor) and its receptors in kidney and bladder carcinomas. *Am J Pathol* **143**: 1255–1262

Brown LF, Berse B, Jackman RW, Tognazzi K, Guidi AJ, Dvorak HF, Senger DR, Connolly JL and Schnitt SJ (1995) Expression of vascular permeability factor (vascular endothelial growth factor) and its receptors in breast cancer. *Hum Pathol* **26**: 86–91

Connolly DT, Olander JV, Heuvelman D, Nelson R, Monsell R, Siegel N, Haymore BL, Leimgruber R and Feder J (1989) Human vascular permeability factor. Isolation from U937 cells. *J Biol Chem* **264**: 20017–20024

Cox DR (1972) Regression models and life-tables. *J R Stat Soc B* **34**: 187–220

Gordon JD, Mesiano S, Zaloudek CJ and Jaffe RB (1996) Vascular endothelial growth factor localization in human ovary and fallopian tubes: possible role in reproductive function and ovarian cyst formation. *J Clin Endocrinol Metab* **81**: 353–359

Harrison-Woolrych ML, Sharkey AM, Charnock-Jones DS and Smith SK (1995) Localization and quantification of vascular endothelial growth factor messenger ribonucleic acid in human myometrium and leiomyomata. *J Clin Endocrinol Metab* **80**: 1853–1858

Kamat BR, Brown LF, Manseau EJ, Senger DR and Dvorak HF (1995) Expression of vascular permeability factor/ vascular endothelial growth factor by human granulosa and theca lutein cells. *Am J Pathol* **146**: 157–165

Krasnow JS, Zeleznik AJ, Berga SL, Yeo KT and Guzick DS (1996) Vascular permeability factor and vascular endothelial growth factor in ovarian hyperstimulation syndrome: a preliminary report. *Fertil Steril* **65**: 552–555

Mattern J, Koomägi R and Volm M (1996) Association of vascular endothelial growth factor expression with intratumoral microvessel density and tumour cell proliferation in human epidermoid lung carcinoma. *Br J Cancer* **73**: 931–934

Nagy JA, Masse EM, Herzberg KT, Meuers MS, Yeo KT, Yeo TK, Sioussat TM and Dvorak HF (1995) Pathogenesis of ascites tumor growth: vascular permeability factor, vascular hyperpermeability, and ascites fluid accumulation. *Cancer Res* **55**: 360–368

National Institute of Health (1994) Ovarian cancer: screening, treatment, and follow-up. *NIH Consensus Statement* **12**: 1–30

Neufeld G, Tessler S, Gitay-Goren H, Cohen T and Levi BZ (1994) Vascular endothelial growth factor and its receptors. *Progress in Growth Factor Research* **5**: 89–97

Neulen J, Yan Z, Raczek S, Weindel K, Keck C, Weich HA, Marmé D and Breckwoldt M (1995) Human chorionic gonadotropin-dependent expression of vascular endothelial growth factor/vascular permeability factor in human granulosa cells: importance in ovarian hyperstimulation syndrome. *J Clin Endocrinol Metab* **80**: 1967–1971

Olson TA, Mohanraj D, Carson LF and Ramakrishman S (1994) Vascular permeability factor gene expression in normal and neoplastic ovaries. *Cancer Res* **54**: 276–280

Plate KH, Breier G, Weich HA and Risau W (1992) Vascular endothelial growth factor is a potent tumour angiogenesis factor in human gliomas in vivo. *Nature* **359**: 845–848

Samoto K, Ikezaki K, Ono M, Shono T, Kohno K, Kuwano M and Fukui M (1995) Expression of vascular endothelial growth factor and its possible relation with neovascularization in human brain tumors. *Cancer Res* **55**: 1189–1193

Senger DR, Peruzzi CA, Feder J and Dvorak HF (1986) A highly conserved vascular permeability factor secreted by a variety of human and rodent tumor cell lines. *Cancer Res* **46**: 5629–5632

Senger DR, Van De Walter L, Brown LF, Nagy JA, Yeo KT, Yeo TK, Berse B, Jackman RW, Dvorak AM and Dvorak HF (1993) Vascular permeability factor (VPF, VEGF) in tumor biology. *Cancer Metastasis Rev* **12**: 303–324

Takahashi A, Sasaki H, Kim SJ, Tobisu K, Kakizoe T, Tsukamoto T, Kumamoto Y, Sugimura T and Terada M (1994) Markedly increased amounts of messenger RNAs for vascular endothelial growth factor and placenta growth factor in renal cell carcinoma associated with angiogenesis. *Cancer Res* **54**: 4233–4237

Takano S, Yoshii Y, Kondo S, Suzuki H, Maruno T, Shirai S and Nose T (1996) Concentration of vascular endothelial growth factor in the serum and tumor tissue of brain tumor patients. *Cancer Res* **56**: 2185–2190

Yeo KT, Wang HH, Nagy JA, Sioussat TM, Ledbetter SR, Hoogewerf AJ, Zhou Y, Masse EM, Senger DR, Dvorak HF and Yeo TK (1993) Vascular permeability factor (vascular endothelial growth factor) in guinea pig and human tumor and inflammatory effusions. *Cancer Res* **53**: 2912–2918

© Cancer Research Campaign 1997